# Host–Virus Interaction: How Host Cells Defend against Influenza A Virus Infection

**DOI:** 10.3390/v12040376

**Published:** 2020-03-29

**Authors:** Yun Zhang, Zhichao Xu, Yongchang Cao

**Affiliations:** State Key Laboratory of Biocontrol, School of Life Sciences, Sun Yat-sen University, Guangzhou 510006, China; zhangyun6@mail.sysu.edu.cn (Y.Z.); wsxzc35@163.com (Z.X.)

**Keywords:** influenza A virus, innate immunity, adaptive immunity

## Abstract

Influenza A viruses (IAVs) are highly contagious pathogens infecting human and numerous animals. The viruses cause millions of infection cases and thousands of deaths every year, thus making IAVs a continual threat to global health. Upon IAV infection, host innate immune system is triggered and activated to restrict virus replication and clear pathogens. Subsequently, host adaptive immunity is involved in specific virus clearance. On the other hand, to achieve a successful infection, IAVs also apply multiple strategies to avoid be detected and eliminated by the host immunity. In the current review, we present a general description on recent work regarding different host cells and molecules facilitating antiviral defenses against IAV infection and how IAVs antagonize host immune responses.

## 1. Introduction

Influenza A virus (IAV) can infect a wide range of warm-blooded animals, including birds, pigs, horses, and humans. In humans, the viruses cause respiratory disease and be transmitted by inhalation of virus-containing dust particles or aerosols [1]. Severe IAV infection can cause lung inflammation and acute respiratory distress syndrome (ARDS), which may lead to mortality. Thus, causing many influenza epidemics and pandemics, IAV has been a threat to public health for decades [2].

The virus is an enveloped, segmented, negative-strand RNA virus, belonging to the *Orthomyxoviriae* family. The eight viral gene segments encode as many as 18 proteins. Besides polymerase basic 1 (PB1), PB1-N40, PB1-F2, PB2, polymerase acid (PA), hemagglutinin (HA), nucleoprotein (NP), neuraminidase (NA), matrix 1 (M1), matrix 2 (M2), nonstructural protein 1 (NS1) and NS2 (also known as nuclear export protein, NEP), new viral proteins were recently uncovered, such as PB2-S1 [3], PA-X (product of ribosomal frameshifting) [4], PA-related proteins PA-N155 and PA-N182 [5], M42 [6], and NS3 [7]. HA, NA, and M2 proteins constitute surface of the IAV virion, where HA is the most abundant surface protein. According to the genetic and antigenic diversity of the HA and NA proteins, IAVs were divided into 18 HA and 11 NA subtypes. H17N10 and H18N11 subtypes were recently identified in bats [8,9].

### 1.1. IAV Viral Proteins

HA is a type I glycosylated protein, which is responsible for virus entry to host cell. Functional HA protein is a homotrimer structurally composed of a stem region and a globular head region in each monomer. The head region bearing N-acetylneuraminic acid (sialic acid, SA) binding pocket is critical for receptor attachment, and contains most antigenic determinants. The stem region undergoing conformational changes is responsible for low pH-triggered membrane fusion [10], and plays an important role in cross protection against heterosubtypic IAV infection [11]. N38 glycan at this region is critical for elicitation of cross-group antibody responses [12]. HA of diverse IAV subtypes that originated from different species presents distinct receptor-binding preference. For instance, human viruses prefer binding to SAs attached to cell-surface-associated α-2,6-linked galactose, whereas avian viruses prefer α-2,3-linked galactose [13,14,15]. Residue substitutions in the receptor-binding site (RBS) of HA is crucial in determining receptor-binding properties [16]. For instance, amino acid substitutions of S138A/G186V/T221P/Q226L within the RBS affected receptor-binding properties of avian H7N9 HA [17], while G186V was reported to be pivotal for the avian-specific strain to acquire human receptor-binding capacity [18].

NA is a type II glycoprotein with neuraminidase (sialidase) enzymatic activity. Each NA tetramer consists of four identical polypeptides, and each polypeptide contains an N-terminal, a hydrophobic membrane domain, a stalk region, and a globular head domain. NA can cleave SA from the mucus, cell surface, and from viral glycoproteins. While HA mediates virion-SA attachment and fusion, NA is responsible for terminal SA residues cleavage [19]. N-glycolyl and O-acetyl modification of SA could reduce binding affinities of both NA and HA [20]. In addition, NA possesses at least two calcium binding sites [21]. Gene analysis of these Ca^2+^ binding sites reveals that they are related to NA thermostability, further suggesting a correlation between NA thermostability and virus adaption [22]. Furthermore, NA is also the major antigenic target of the host humoral immunity, and NA-specific antibodies function in limiting virus egress via interfering with the sialidase activity have drawn wide attention for development of antiviral therapies [23,24].

The viral ribonucleoprotein complex (vRNP) is a rod-shaped structure composed of multiple copies of NP and a single trimeric RNA-dependent RNA polymerase complex (PB1, PB2, and PA) associated with viral genomic RNA [25]. NP mediates nuclear import of the vRNP complex, the PB1 subunit has the catalytic polymerase activity, the PB2 subunit contributes to cap binding, and the PA subunit is required for cleavage of the capped oligonucleotides. The complex is required for the transcription and replication of the viral genome [26]. The structure, functions, and modulation of the IAV RNA polymerase complex were further discussed by Te Velthuis and Fordor [27]. However, the mechanism of vRNP assembly remains largely unknown and several host proteins were reported to be involved during IAV infection [28,29,30]. Recently, eleuthe roside B_1_ was demonstrated to be able to inhibit the vRNP in vitro [31].

M1 and M2 are encoded by the *M* gene on segment 7 of the viral genome [32]. The conserved M1 bears a positively charged nuclear localization sequence (NLS) motif RKLKR, which is essential for membrane binding, virus replication, nuclear export of the vRNPs, and self-polymerization [33]. M2 is a type III integral transmembrane proton channel, structurally containing three domains: an amino-terminal ectodomain, a transmembrane domain, and a cytoplasmic domain [34]. It is essential for uncoating the vRNP complex within the endosome, the process of which is essential for viral entry [35]. Moreover, in its retrograde migration route from the membrane to the cytoplasm, M2 was shown to prevent endosome fusion with the lysosome, suggesting new roles of the protein in the host cytoplasm after infection [36].

NS1, structurally composed of an N-terminal dsRNA binding domain and a C-terminal effector domain, plays important roles in control of viral RNA synthesis, viral mRNA splicing, and virus particle morphogenesis during the virus life cycle [37,38]. It is also considered as the key factor to antagonize host immune system [39], since it could inhibit activation of retinoic acid-inducible gene-I proteins (RIG-I), interferon-inducible RNA-dependent protein kinase (PKR), and 2′-5′-oligoadenylate synthetase of the innate immunity via blocking nuclear export of host messenger RNAs (mRNAs) [40,41,42]. The mechanism underlying is revealed by determination of crystal structure of the NS1 and nuclear RNA export factor 1-nuclear transport factor 2-related export protein 1 (NXF1-NXT1) complex that NS1 could prevent mRNA export through the nuclear pore complex via binding of NXF1-NXT1 to nucleoporins [43]. Conserved RNA secondary structures at RNA positions 82-148 and 497-564 are essential for NS1 protein expression, thus affecting viral reproduction and virus–host interaction processes [44]. A detailed description of structure and function of the NS1 was reviewed by Han and his colleagues [45]. NS2, also known as NEP, contains a nuclear export signal and is responsible for chromosome region maintenance 1 (Crm1)-dependent nuclear export of RNPs in the infected cells [46,47]. Crm1 contains leucin-rich nuclear export signals (NES1 and NES2) [47,48], which are important for the NS2Crm1 interaction [46,48]. In addition, interaction of CHD3 and NES1 was identified to be important in the NS2-mediated vRNP nuclear export [49].

### 1.2. Antigenic Shift and Antigenic Drift

Genome reassortment often occurs when two or more IAV strains infect a single host cell. This phenomenon is refereed as antigenic shift, resulting in novel progeny viruses with new HA and/or NA that are immunologically naïve to the human immune system [50,51]. On the other hand, lack of proof-reading function of viral RNA polymerase results in accumulated point mutations during successive replication of the viral genome. This phenomenon is refereed as antigenic drift [52]. The two mechanisms provide IAVs the ability to evolve rapidly as well as to evade host immunity, thus resulting in generation of viral strains which are highly pathogenic and/or can cross species barriers.

Since the “Spanish influenza” pandemic in 1918–1919, despite application of updating licensed vaccines and drugs, seasonal and pandemic influenza still cause millions of infection cases each year and remains a worldwide public-health concern. Most seasonal IAV infections are minimally symptomatic, while severe infection causes damage to host immune responses, thus resulting in lung inflammation and injury [53]. The virus itself, IAV–host interaction, as well as a need for next-generation IAV prevention and control strategies attract extensive attention worldwide. Understanding the virus–host interaction can facilitate investigation of antiviral measures. Therefore, in the current review, we summarized and discussed recent development in our understanding of IAV–host immune interaction and how IAV antagonizes different immune responses to achieve a successful infection.

## 2. Influenza A Virus Host Adaption and Life Cycle

### 2.1. Influenza A Virus Host Switch Events

IAVs can infect a broad spectrum of host species, including both wild and domestic birds, as well as many mammalian species. The virus is capable of interspecies transmission to new species. However, no interspecies transmission of the bat IAVs has been reported so far [54]. Furthermore, the high frequency of mutations and recombination increases the risk of IAV adaptation in humans. Besides three pandemic subtypes (H1N1, H2N2, and H3N2), other subtypes, including H5N1, H5N6, H7N7, H7N9, N9N2, and H10N8 could cross the species barrier and cause human infections [55,56,57,58]. Several effect factors are essential in IAV host switch events, including the receptor-binding properties of HA [16], as well as cellular receptors [59,60,61,62]. Long and his colleagues summarized the role of host factors in IAVs adaption to humans, and the review is recommended here for further reading [63].

Noteworthy is the fact that most phylogenetically diverse IAVs with different origins could successfully replicate in swine [64]. Since pigs have both SA α-2,6 and SA α-2,3 galactose receptors [65], they can serve as a suitable mixing reservoir for both human and avian IAVs, thus raising global concern on periodic zoonotic infections. Take the emergence of influenza A (H1N1) pdm09 (pH1N1) and influenza A (H3N2) A/Canada/1158/2006 strains for instance, both strains are swine-origin IAVs and were the consequence of adaption and reassortment of several swine lineages [66,67]. Furthermore, some genes of these strains originated from avian IAVs [68].

With the development of gene sequencing technology, machine learning (ML) facilitated with large genomic datasets are used in prediction about sequence changes in newly invaded viruses from other animal hosts, take the “Batch-Learning Self-Organizing Map (BLSOM)” method for instance [69]. ML is also applied in characterization of distinct host tropism protein signatures [70], and prediction of amino acid changes for interspecies transmission [71]. These studies provided measures in identification of potential high-risk strains. In addition, the nucleotides and dinucleotide compositions of viruses play important roles in prediction of viral host species [72]. Combining gene sequencing technology and ML methods, researchers applied large IAV genomic datasets to analyze species selection bias of IAV mono-/dinucleotide composition and predict human-adaptive swine or avian IAVs [73,74]. The application of multi-disciplinary subjects would provide useful information for prediction of pandemic influenza.

### 2.2. Life Cycle of Influenza A Virus

In general, the life cycle of the IAV is generally divided into four steps: virus entry into the host cell, transcription and replication of the viral genome, assembly, and virus budding. Though alveolar epithelial cell is the primary target cell for IAVs, different IAV subtypes have different patterns of viral attachment (PVA). For human IAVs, Alveolar type II epithelial cells, as well as immune cells such as alveolar macrophages and dendritic cells, are major target cells for an established infection [75,76]. Two seasonal IAVs and pandemic H1N1 virus, preferred to attach to ciliated epithelial cells and goblet cells in the upper respiratory tract (URT), and avian IAVs, take H5N1 for instance, attached seldom to these cells [77]. In the lower respiratory tract (LRT), human IAV H1N1 and H3N2 attached to more cell types than avian IAV H5N1, a highly pathogenic avian IAV (HPAIV) strain. However, H5N1 could bind to type II pneumocytes [78]. Considering the fact that metabolism in the type II pneumocytes is quite active, infection of HPAIVs is more likely to cause severe pneumonia [78]. Other research on low pathogenic avian IAVs (LPAIVs), which generally do not cause severe pneumonia, showed that these viruses usually attach to human submucosal gland cells, thus can be cleared by the mucus [79].

IAV infection starts from recognition of SA by HA protein, though in vitro research claimed that these N-linked glycans were not essential for virus entry [80]. The cleavage of HA precursor protein HA0 into HA1 (containing receptor binding domain) and HA2 (containing fusion peptide) in low pH environment during HA transport is critical for virion internalization [81]. Some research showed that type II transmembrane serine protease such as transmembrane protease serine 2 (TMPRSS2), human airway trypsin-like protease (HAT), transmembrane protease serine 4 (TMPRSS4), Homo sapiens serine protease DESC1 and Homo sapiens transmembrane protease, serine 13 (MSPL) can cleave human and avian IAV HA proteins at an arginine residue [82]. In addition, for avian IAVs, HA0 of HPAIVs can be cleaved by subtilisin-like protease, while that of LPAIVs is cleaved by trypsin-like proteases [83] or thrombin [84]. Therefore, in avian IAVs, the cleavage sites are considered to be the major determinants for virus virulence [85], and RNA folding in the cleavage region could be an important factor for virulence determination [86,87].

Proteins in the vRNP complex contain different nuclear localization signals (NLSs), thus helping the vRNP complex to enter the host cell nucleus via active transport, take the Crm1-dependent pathway for instance [88]. The acidic environment of the endosome also activates M2 ion channel, hence acidifies the viral core, resulting in entrance of vRNP complex into the host cell [34]. Replication of viral genome does not require a primer but a full-length complementary RNA (cRNA), which is essential for the newly formed vRNP complex. The viral RNA polymerases first bind to the 3′ end and the 5′ end of the segmented viral RNA and cRNA, respectively, then start replication with the help of the 5′ cap of host pre-mRNAs via a PB1-PB2-mediated “cap snatching” mechanism [27,89]. The conserved segment-specific nucleotides at the 3′ and 5′ ends of the viral genome could modulate genome expression and replication during infection [90]. In addition, dephosphorylation at a specific position of the H1N1 NS1 protein results in attenuated virus replication [91].

Mature viral mRNAs are transported to the cytoplasm by a “daisy-chain” complex and translated subsequently [88,92]. New synthesis of HA occurs on the rough endoplasmic reticulum (ER). Glycosylation and palmitoylation of the protein are completed later in the Golgi [93,94]. After synthesis and maturation of NA and M2 proteins, the trans-Golgi network (TGN), together with coat protein I (COPI) complex and GTPase Rab proteins, transport the newly synthesized HA, NA, and M2 proteins to the apical plasma membrane (PM). These proteins then assemble with viral genomic segments. The virions are finally closed and M1 and M2 proteins mediate virion budding from the apical side of the cells [28,95,96,97,98]. NA protein cleavages the SA residues, which allows the virions to be released from the plasma membrane [99].

Since IAV has a relatively small genome, host machinery is required in order to accomplish the viral life cycle. To uncover host dependency factors that are necessary for IAV replication, numerous large-scale RNA interference (RNAi) screens and genome-wide CRISPR/Cas 9 screen were performed [29,100,101,102,103]. For instance, SON DNA binding protein was important for IAV virion trafficking in an early infection stage and CDC-like kinase 1 facilitated AIV replication [29]. USP47 facilitated viral entry, whereas TNFSF12 (APRIL) and TNFSF12-TNFSF13 (TWEPRIL) helped with viral replication [101]. Using genome-wide CRISPR/Cas9 screen, several genes of sialic acid biosynthesis and related glycosylation pathways were involved with H5N1 infection [102], and WDR7, CCDC115, and TMEM199 were essential for viral entry and regulation of V-type ATPase assembly [103]. Furthermore, single-cell transcriptome sequencing (RNA-seq) was applied to explore host–virus interactions, revealing a correlation between defective viral genomes and virus-induced host transcriptional programs [104]. These data provide valuable information for developing host-targeted therapeutics.

## 3. Host Immune Responses Against IAV Infection

Host immune system functions immediately after detection of the virus. Host mucosal immune system (MIS), induced after virus invasion, serves as the first line to prevent IAV from adhering to the susceptible cells. In the URT, mucosal response is induced in the naso-associated lymphoid tissues (NALT), while in the LRT, it occurs in bronchus-associated lymphoid tissues (BALT). Host innate immunity, including phagocytic cells, interferons (IFNs), proinflammatory cytokines, etc., applies multiple mechanisms in defending IAV infection [105]. Host adaptive immunity, mediated by B lymphocytes and T lymphocytes, together with other immune mechanisms, reacts specifically to neutralize and eliminate the virus. On the other hand, to establish a successful infection, IAVs also employ a plethora of strategies to avoid being detected or being cleared by the host immunity. Notable strategies include regulation of IFN signaling [106], inhibition of cytokine expressions [107,108], modulation of apoptosis [109,110,111], interference of autophagy [112], and effects on antibody production [113]. The IAV–host immunity interaction was summarized by several reviews [114,115].

### 3.1. Immune Cells Involved in IAV Infection

Upon detection of infection, innate effector cells, including natural killer (NK) cells, neutrophils, and dendritic cells (DCs), etc., are recruited to the infected sites. NK cells are large granular lymphocytes, making up 10% of the resident lymphocytes in the lung. After recruitment from the blood, NK cells interact with DCs and macrophages to secret various cytokines and restrict infection via lysis of the IAV-infected cells. The lysis process is mediated by interaction between NK receptors p46 (most NKp46) and IAV HA protein expressed by the infected cell [116,117]. Interestingly, liver NK cells other than lung NK cells possessed a memory phenotype to protect mice against subsequent IAV infection, though the lung NK cells are important in control of primary IAV infection [118]. However, NK cells are also shown to exacerbate IAV pathology, since depletion of NK cells led to increased resistance to high dose H1N1 infection in mice [119,120]. The contribution of NK cells to anti-IAV defense in mouse models was later shown to be strain and dose dependent. In addition, the host genetic background also played an important role [121].

Neutrophils are key innate immune cells recruited to infection sites by cellular migration through vascular endothelium. They function in clearance of pathogens via phagocytosis, producing extracellular traps, and degranulation [122]. In addition, they also regulate adaptive immunity via guiding influenza specific CD8^+^ T cells to the infection sites [123].

The function of dendritic cells (DCs) is to monitor invading pathogens. After IAV infection, the conventional DCs migrate from lung to lymph nodes through interaction between CCR7 and its ligand, and present antigens to T cells [124,125]. One study based on a mouse model showed that, during IAV infection, immature and mature DCs were specialized in IAV HA processing, since both types of DCs could present one epitope of H1N1 HA (HA amino acids 107–119), whereas another epitope (HA amino acids 302–313) could only be processed by mature DCs [126]. The complex role of DCs in initiation of robust immunity against IAV infection is reviewed by Waithman and Mintern [127].

T cells and B cells are critical components in adaptive immunity against IAV infection. CD8^+^ T cells differentiate into cytotoxic T lymphocytes (CTLs) and defend IAV infection via producing cytokines and effector molecules, and cytotoxic effects (i.e., lysis) of infected cells mediated by MHC class I. CD4^+^ T cells target IAV-infected epithelial cells through binding with MHC class II molecules and contribute to B cell activation thus consequently promote antibody production. The activation of T cells and B cells in IAV infection will be exposited in Section 3.5.

### 3.2. Activation of Innate Immunity in IAV Infection

The reaction of innate immunity is nonspecific. It is triggered by recognition of pathogen associated molecular patterns (PAMPs) via host pathogen recognition receptors (PRRs). Toll-like receptors (TLRs), retinoic acid-inducible gene-I proteins (RIG-I), and NOD-like receptors are common PRRs, the activation of which leads to activation of innate immune signaling and further production of cytokines as well as other antiviral molecules.

Toll-like receptors are responsible for sensing pathogens at cell membranes, endosomes, and lysosome [128]. TLR3 and TLR7 are shown to be involved in IAV detection at endosomes [105]. TLR3 recognizes double stranded RNA (dsRNA) which may be released by cellular stress and cell death [105] and unidentified RNA structures in phagocytosed cells infected with IAVs [129]. In macrophages and dendritic cells, TLR3 interacted with TIR-domain-containing adapter, then activated the serine-threonine kinase IκKε (IKKε) and TANK binding kinase 1 (TBK1) to phosphorylate interferon regulatory factor 3 (IRF3), the process of which further led to expression of IFN-β [130]. In addition, an over-reacting TLR3 activation promoted IAV pathogenesis, which could be reduced by a single-stranded oligonucleotide (ssON) functioning as a TLR3 inhibitor, resulting in restrained viral loads both in vitro and in vivo [131]. TLR7 recognizes single stranded RNA (ssRNA). In plasmacytoid dendritic cells (pDCs), after activation of TLR7 during IAV infection, IRF7 or nuclear factor kappa-light-chain-enhancer of activated B cells (NF-κB) were activated via myeloid differentiation factor 88 (MyD88) to induce type I IFNs [132]. In avian macrophages, activation of TLR7 produced pro-inflammatory molecules such as interleukin (IL)-1β [133]. In addition, in mouse models, TLR7 played an important role in activation of NK cells [134]. It was also shown to be involved in development of adaptive immunity to prevent IAV infection [135,136].

RIG-I recognizes ssRNAs and transcriptional products of IAVs, which triggers activation of the caspase activation and recruitment domains (CARDs) via dephosphorylation or ubiquitination by E3 ligases, resulting in activation of transcription factors including IRFs and NF-κB [137]. OTUB1 played an essential role in regulation of RIG-I [138]. In addition, melanoma differentiation-associated gene 5 (MDA5) was also involved in sensing transcriptional products of IAVs in the cytoplasm [139].

For NOD-like receptor family, pyrin domain containing 3 (NLRP3) and NLR apoptosis inhibitory protein 5 were activated after IAV infection [140]. IAV M2 ion channel and PB1-F2 were involved in activation of NLRP3 inflammasome and stimulate IL-1β secretion subsequently [141,142]. The role of the NLRP3 inflammasome in regulation of anti-IAV responses is discussed in detail by Sarvestani and his colleagues [143]. Delayed oseltamivir and sirolimus combined treatment could suppress NLRP3 inflammasome mediated secretion of IL-1β and IL-18, resulting in attenuation of H1N1-induced lung injury [144].

### 3.3. The Host Interferon (IFN) Response in IAV Infection

After detecting viral components, transcription factors including NF-κB and IRFs are activated, leading to transcription of IFNs and pro-inflammatory cytokines. IFNs bind to receptors, resulting in upregulation of multiple interferon-stimulated genes (ISGs) [145]. It is well known that type I IFNs (IFN-α and IFN-β) and type III IFNs (IFN-λ 1-4) play critical roles in antiviral responses. Mice failed to restrict non-pathogenic IAV when both type I and type III IFN receptors were knocked out [146].

The expressed IFNs consequentially bind to different receptors. Type I IFNs interact with IFN-α/β receptors (IFNAR), whereas type III IFNs interact with IFN-λ receptors (IFNLR). Janus kinase-signal transducer and activator of transcription (JAK-STAT) signaling pathway is then activated, resulting in transcription of numerous IFN-stimulated genes (ISGs) [147,148]. Though IFN-λs share many characteristics such as expression patterns, signaling pathways, etc. with type I IFNs, they are the first IFNs produced at the infected epithelial sites to block virus spread [149]. Furthermore, IFN-λs served an important role in programming DCs to direct effective T cell immunity against IAV infection [150].

**Figure 1:**
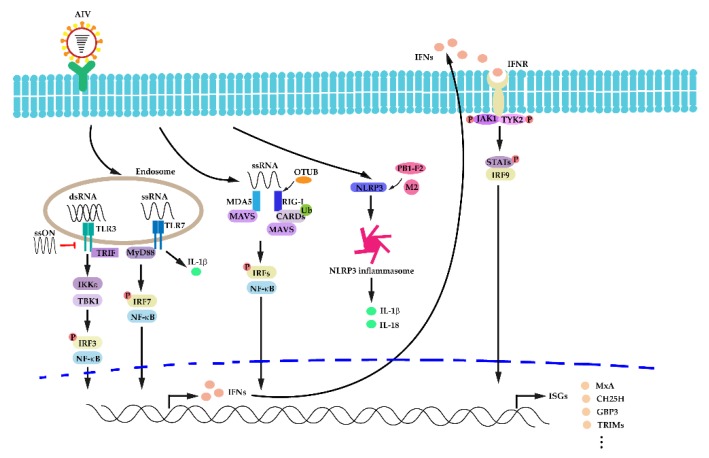
Schematic diagram for activation of innate immune responses and interferon (IFN) signaling pathway after influenza A virus (IAV) infection. Intracellular detection conducted by pathogen recognition receptors (PRRs), including retinoic acid-inducible gene-I proteins (RIG-I), melanoma differentiation-associated gene 5 (MDA5), and toll-like receptors (TLRs), activate transcription factors such as IRFs and nuclear factor kappa-light-chain-enhancer of activated B cells (NF-κB), resulting in expression of IFNs and interferon-stimulated genes (ISGs).

ISGs encode various antiviral proteins functioning in different ways to defend IAV infection. For instance, MxA GTPase from the Mx family could retain viral genome from entry to the cytoplasm via blocking the function of IAV NP. In addition, in vitro research found that avian IAVs were more sensitive to MxA than human IAVs [151,152]. Cholesterol 25-hydroxylase (CH25H) were identified to block IAV entry via altering the cellular membrane properties to interfere with viral fusion, and amplified the activation of immune cells [153]. Guanylate-binding protein 3 (GBP3) of IFN-inducible GTPases inhibited IAV replication via binding to the viral polymerase complex [154]. Members of the tripartite motif-containing (TRIM) family are also involved in cellular anti-IAV processes. For instance, TRIM14 could interact with IAV NP for ubiquitination and proteasomal degradation, thus restricting IAV replication in a type I IFN and NF-κB independent manner [155]. TRIM22 degraded IAV NP via polyubiquitination, thus resulting in inhibition of IAV infection [156]. TRIM 25 regulated the re-localization of RIG-I and was responsible for RIG-I ubiquitination as well as RIG-I-mediated IFN production [157]. TRIM32 recognized IAV PB1 protein and reduced its polymerase activity [158]. TRIM41 targeted NP for ubiquitination and degradation in vitro [159]. For further reading on other ISGs, several reviews regarding IFN responses during IAV infection are recommended here [160,161]. A general description of activation of innate immunity and IFN signaling pathway after IAV infection is illustrated in Figure 1.

In order to counter IFN-stimulated antiviral proteins, IAV viral proteins apply multiple strategies. For instance, HA protein was shown to trigger ubiquitination of IFNAR to attenuate the type I IFN signaling pathway [162]. The follow-up work showed that poly (ADP-ribose) polymerase 1 (PARP1) functions as an interacting partner of HA protein to mediate the HA-induced IFNAR degradation [163]. NS1 is the most important IFNs antagonist protein via mechanisms including inhibition of the TRIM25-mediated RIG-I ubiquitination, suppression of protein kinase R (PKR), phosphorylation of IκB kinases (IKK) α and β in the NF-κB pathway, interruption of the phosphorylation of STAT1, STAT2, and STAT3 [39,115], and degradation of OTUB1 [138]. Phosphorylation of NS1 is crucial for its function of antagonizing IFN-β expression, since dephosphorylation at position 73 and 83 of the protein induced a high level of IFN-β [91]. Non-structural protein PB1-F2, identified from a+1 open reading frame (ORF) of PB1 gene segment [164], is multifunctional in deregulation of type I interferon [165,166]. It counteracted RLR-mediated activation of IFN pathway not only by targeting mitochondrial MAVS [115,165,167], but also by binding to the DEAD-box helicase DDX3 to induce proteasome-dependent degradation [166]. Furthermore, PB1-F2 interacted with mitochondrial Tu translation elongation factor (TUFM) to mediate formation of autophagosome, thus inducing complete mitophagy, which is critical for MAVS degradation [167]. Novel PA-X protein could also modulate innate immune responses. A review regarding the function of NS1 and PA-X proteins in antagonizing host innate immunity is recommended here [114].

### 3.4. Autophagy

Though autophagy is essential for cellular metabolism and homeostasis, it also plays important roles in innate immune responses against pathogen infection. For cellular homeostasis, the mTOR pathway is one of the most conserved autophagic pathways. The mTOR complex 1 (MTORC1) negatively regulates the ULK1 kinase activity, thus affecting the autophagy induction [168]. c-Jun N-terminal protein kinase 1 (JNK1) disrupts the Bcl-2/Beclin-1 complex through phosphorylation, thus regulating the autophagy induction [169,170]. JNK1 is also reported to upregulate Beclin-1 expression through phosphorylation of transcription factor c-Jun in vitro [171].

In contrast to the autophagic pathways for cellular metabolism and homeostasis, less is known about autophagosome formation after IAV infection [172]. To restrict infection of multiple viruses including IAVs, TRIM23 is essential to mediate autophagy via its RING E3 ligase and ADP-ribosylation factor (ARF) GTPase activity [173]. Beclin-1 and TUFM-regulated autophagy also inhibited IAV replication [112]. In HeLa cells and A549 cells, IAV infection activated JNK1 to induce autophagosome formation and TGF-β-activated kinase 1 might contribute to the process [174,175]. Furthermore, autophagy was involved in maintaining memory B cells to counteract IAV infection [176].

IAV also utilizes autophagy to complete its life cycle. NS1 protein is proposed to suppress JNK1-mediated autophagy induction [174]. M2 could also block autophagosome maturation and mediate microtubule-associated protein 1 light chain 3 (LC3)-bound membrane redistribution, thus allowing filamentous budding of IAV [177,178,179]. Circ-GATAD2A (GATA zinc finger domain containing 2A), induced by IAV infection, could inhibit autophagy and promote IAV replication [180]. For a comprehensive reading on IAV-induced apoptosis, a review is recommended here [181].

### 3.5. Adaptive Immunity against IAV Infection

Upon detection of IAVs, DCs trigger production of IFNs and cytokines, which in turns assist maturation of the DCs into antigen presenting cells (APCs), and initiate T cell immune responses. Through the activation of Ag-bearing DCs, naïve CD4^+^ T cells differentiate into Th1, Th2, Th7, regulatory T cells (Treg cells), follicular helper T cells, and killer cells. Th1 and follicular helper T cells are the most abundant CD4^+^ T helper cells. They can secret antiviral cytokines, regulate CD8^+^ T cell differentiation, promote B cell activation, and maintain immunological memory [182,183]. Th17 cells induced pulmonary pathogenesis and could decrease mortality of IAV-infected mice [184,185]. In addition, γδ T cells, expanding in the late stage of IAV infection with a T cell receptor (TCR)-independent manner, could efficient eliminate IAV-infected airway epithelial cells, resulting in lower viral titers [186]. New surrogate markers CD49d and CD11a were used to explore the kinetics of IAV-specific CD4 T cells responses, revealing endogenous CD4 T cell response to primary IAV infection is predominantly composed of T-bet+ cells [187].

CD8^+^ T cells are major components for virus clearance in adaptive immunity. After activated by DCs, CD8^+^ T cells undergo rapid expansion, differentiation, and migration to the infected sites. In general, to establish effective primary cytotoxic T lymphocyte (CTL) responses, CD4^+^ T cells play an essential role, with a mouse model as an exception [188]. CTLs produce cytotoxic granules containing perforin and granzymes (GrA and GrB) to induce apoptosis and interrupt IAV replication [189]. In addition, CTLs produce cytokines, such as TNF, FASL, and TRAIL, which recruit death receptors to induce apoptosis [190]. In addition, IL16 deficiency enhanced the Th1 and CTL responses upon IAV infection [191]. Furthermore, as CD8^+^ cells could last for two years in murine models, IAV-specific memory CTLs reacted specific to epitopes in conserved IAV proteins [192]. In the nasal epithelia, they could prevent the spread of the virus from the URT to the lung [193]. To establish memory CD8^+^ T cells, autophagy plays an important role [194], while the function of CD4^+^ T cells in memory CTL responses is “context-dependent”. A recent study showed that CD4+ T cells promoted IAV-specific CTL memory at the initial priming stage of viral infection [195]. Grant and her colleagues summarized and discussed the importance of CD8^+^ T cell immunity against IAVs [192], and this review is recommended for further reading.

**Figure 2:**
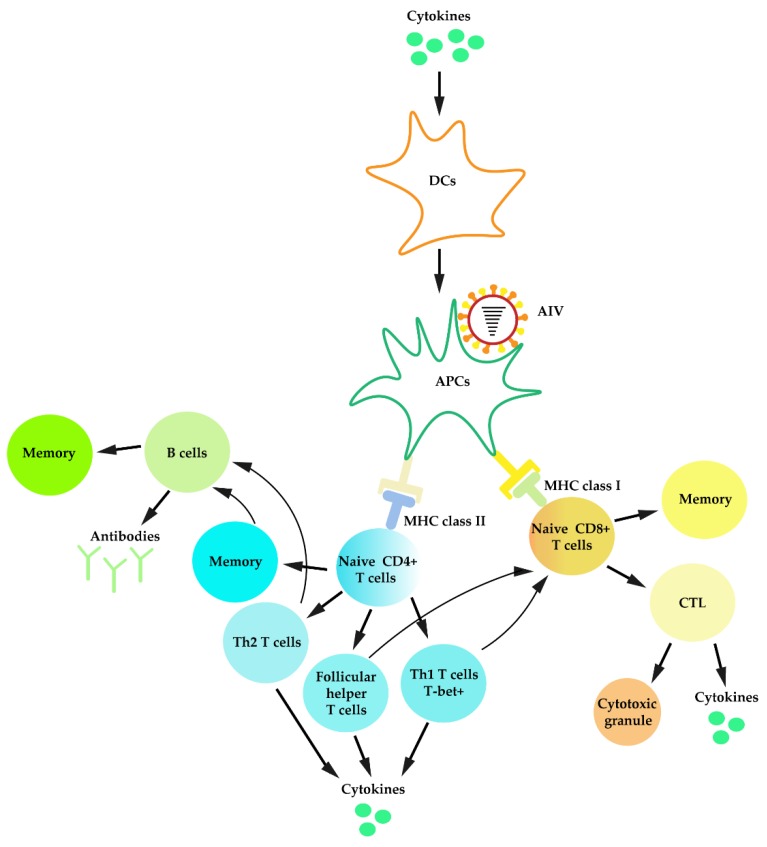
Schematic diagram for adaptive immune responses against primary IAV infection.

With the help of CD40 ligand (CD40L), CD4^+^ cells contribute to B cell activation [183]. With the help of memory T cells, naïve B cells could reduce morbidity and promote recovery on heterosubtypic infection [196]. For different types of antibodies, IgG could inhibit pathogenesis, while IgA functions in blocking IAV transmission [197]. In addition, IAV-specific antibody-dependent cell-mediated cytotoxicity (CDCC) also plays a role in cross-protection against IAV infection. A general description of adaptive immunity against primary IAV infection is illustrated in Figure 2.

Antigenic shift and drift, resulting in reassorted and mutated HA and/or NA, are responsible for AIV escaping from host immunity [50,51,52]. Furthermore, additional glycosylation on H5 HA could also induce virus escape from neutralizing antibodies [198].

### 3.6. Apoptosis against IAV Infection

Apoptosis represents programmed single cell death that occurs in cell physiological remodeling, cell proliferation, or immune response to invading pathogens [199]. Besides prototypical changes, cells undergoing apoptosis can be detected through DNA and biochemical assays, take the TUNEL and in situ end-labeling (ISEL) techniques for instance. Two primary pathways are involved in activation of apoptosis: the intrinsic or mitochondrial pathway, and the extrinsic or death receptor pathway.

#### 3.6.1. The Intrinsic and Extrinsic Apoptosis Pathway

The intrinsic pathway is also known as “the mitochondrial pathway”, which operates in response to various intracellular stress. Several factors such as nitric oxide (NO), cytochrome c, and second mitochondria-derived activator of caspases (SMAC) can activate this pathway, and the key player of this pathway is proteins in the bcl-2 family, which are activated by stress signals and then release apoptotic factors via destabilizing the mitochondrial membrane [200,201], resulting in release of mitochondrial cytochrome c. Cytochrome c then binds to apoptosis protease activating factor-1 (APAF-1) and forms a complex with pro-caspase 9 (then cleaved into caspase 9), the function of which is to cleave its effector pro-caspase 3 [202]. In addition, SMAC, localizing in the cytosol, could initiate activation of caspase 9 via blocking the activity of IAP [203]. The extrinsic pathway is regulated by extracellular ligands acting on transmembrane “death receptors”: the first apoptosis signal (FAS) receptor-FAS ligand (FASR/FASL) and the TNF-αTNF receptor 1 (TNFα/TNFR1) [199]. In the FASR/FASL model, FAS ligand binds to its receptor FASR [204], forming the death-inducing signaling complex (DISC) with pro-caspase 8, resulting in activation of caspase 8 and downstream activation of other caspases (caspase-3, caspase-6, and caspase-7) [199]. In the TNFα/TNFR1 pathway, TNFR1-associated death domain protein (TRADD) is activated after binding of TNFα to TNFR1, leading to recruitment of FADD and receptor interacting protein (RIP) [205]. FADD then associates with pro-caspase 8 to form the DISC, resulting in activation of caspase 8 and apoptosis.

#### 3.6.2. Apoptosis after IAV Infection

During IAV infection, viruses modulate host apoptotic responses in a time-dependent manner [206]. For instance, in order to earn enough time for replication and virion formation, IAV inhibited apoptosis via upregulating the anti-apoptotic phophoinositide-3-kinase-protein kinase B (PI3K-AKT) pathway at the beginning of infection. However, in the later phase of infection, the virus suppressed this pathway to upregulate the pro-apoptotic p53 pathway, thus allowing successful release of virions [207].

Several viral proteins are involved in regulation of host apoptosis. NP protein induces host apoptosis to favor viral replication through interaction with ring finger 43 (RNF43) [208], apoptotic inhibitor 5 (API5) [209], or clusterin [111]. PB1-F2 also induced apoptosis and promoted viral replication through dysregulating mitochondrial potential [206]. Furthermore, M1 promoted apoptosis by binding to heat shock protein 70, thus activating caspase and the subsequent apoptosis [210]. In addition, NS1 expression was reported to induce apoptosis in MDCK and HeLa cells [211]. However, mutant IAV lacking the NS1 gene could induce apoptosis in cultured cells [212]. The function of NS1 in inhibiting apoptosis may be explained by its ability to inhibit type I IFN [213,214]. These data demonstrate sophisticated mechanisms of IAV in regulating host apoptosis. Furthermore, the role of these viral proteins in apoptosis suggests that these proteins may present suitable targets for anti-IAV therapies. A comprehensive review on influenza A virus-induced apoptosis discussed by Ampomah and Lim is recommended here [181]. In addition, recent in vitro research found that apoptosis was induced at early IAV infection stage, while later the cell death pathway was shifted to pyroptosis. The switch process was promoted by the type I IFN-mediated JAK-STAT signaling pathway through expression of the Bcl-xL gene [215].

## 4. Perspectives

During IAV infection, multiple immune systems coordinate together to protect the host. Accordingly, viruses antagonize the immune system through multiple measures to establish a successful infection. Considering the high frequencies in genome mutations and recombination, vaccination is the most effective way to defend against the viruses via inducing cross-protective antibodies and/or enhancing immune responses. Several studies in vaccine development have tried to enhance host immune responses. For instance, vaccine candidate containing HA targeted to chemokine receptor (porcine MIP1α) was shown to enhance T cell responses, resulting in a strong and cross-reactive cellular immunity in vaccinated pigs [216]. Another example is an attempt to intranasally administer a polyanhydride nano vaccine (IAV-nanovax), which could promote robust lung-resident germinal center (GC) B cells with lung-localized IAV-specific antibody responses as well as lung-resident memory CD4^+^ and CD8^+^ T cell responses [217].

For anti-IAV drugs, currently, NA inhibitors (Relenza^TM^ and Tamiflu^TM^) are applied clinically as anti-influenza drugs [218]. These drugs inhibit the activity of NA by preventing viral budding [21]. In addition, cap-dependent endonuclease inhibitor (Baloxavir Marboxil) targeting PA is also applied against influenza A and B virus infection [219]. Our progressing understanding of the IAV life cycle of the virus and IAV–host interaction could contribute to anti-influenza drug design.

Since the recognition of HA protein to SA linked glycoproteins is the first step in IAV infection, effective blocking of the interaction between viral HA and SA receptor serves as a favorable target in drug design [220,221]. Favipiravir, a nucleotide analogue that selectively inhibits the RNA-dependent RNA polymerase, is licensed in Japan to be applied against emerging influenza viruses resistant to other antivirals [222,223]. Oleanolic acid (OA), a kind of pentacyclic triterpene natural product, and its analogues, as well as its derivatives, were shown to bind to HA, thus blocking the attachment of IAVs to MDCK cells [224,225,226]. PVF-tet is a peptide-based HA inhibitor, which was shown to sequester HA into amphisome (fusion of late endosome with autophagosome) and protected mice from the lethal IAV infection [227].

New effective drugs targeting the polymerase would be a promising strategy against IAV infection, since they would directly reduce or eliminate viral replication. Numerous sites, including the cap-binding site [228], the endonuclease [229,230], and PA-PB1 inter-subunit interface [231] can serve as potential targeting sites for new drug design. Coumarin compounds, including Eleutheroside B_1_, Isofraxidin, Fraxin, Esculetin, Fraxetin, and Scoparone, were investigated for their antiviral and anti-inflammatory activities against influenza virus in vitro [31].

Other candidates, such as Naproxen, a non-steroidal anti-inflammatory drug, was shown to target NP protein at residues F209 and Y148, thus antagonizes the CRM1-mediated nuclear export of NP. It is suggested to have a broad-spectrum anti-influenza activity [232]. Verdinexor (KPT-335), a novel orally bioavailable drug, blocks CRM1-mediated nuclear export of NP and repress NF-κB activation, thus reducing cytokine production and eliminating virus-associated immunopathology [233]. For further reading on candidate anti-IV therapeutics, a review summarized by Davidson is recommended here [234].

With the increasing knowledge obtained through massive investigations on host immunity against IAV infection, promoting host immune responses not limited to antibody enhancement would have good prospects not only for vaccine design, but also for development of novel antiviral agents.
